# Survival Outcomes and Prognostic Predictors in Patients With Malignant Struma Ovarii

**DOI:** 10.3389/fmed.2021.774691

**Published:** 2021-12-23

**Authors:** Sijian Li, Shujun Kong, Xiaoxue Wang, Xinyue Zhang, Min Yin, Jiaxin Yang

**Affiliations:** ^1^Department of Obstetrics and Gynecology, Peking Union Medical College Hospital, Chinese Academy of Medical Sciences, Peking Union Medical College, National Clinical Research Center for Obstetric & Gynecologic Diseases, Beijing, China; ^2^Department of Obstetrics and Gynecology, The Affiliated Yantai Yuhuangding Hospital of Qingdao University, Yantai, China

**Keywords:** malignant struma ovarii, thyroid carcinoma, treatment, survival outcomes, prognostic factors

## Abstract

**Background:** Malignant struma ovarii (MSO) is an extremely rare ovarian malignant tumor and there is limited data on the survival outcomes and prognostic predictors of MSO. The objectives of this study were to investigate the disease-free survival (DFS), overall survival (OS), and disease-specific survival (DSS) rates of patients with MSO, and also evaluate the prognostic factors in this population.

**Methods:** A retrospective study was conducted and 194 cases of MSO were selected. DFS was assessed by the logistic regression, OS by the Kaplan–Meier method, and DSS was evaluated by the Cox regression.

**Results:** The median age of these patients was 46.0 years; 142 cases (73.2%) were confined to the ovary and 52 cases (26.8%) had extraovarian metastasis at the initial diagnosis of MSO. During the follow-up, 75.3% of these patients showed no evidence of disease and 18.0% were alive with disease. Only 13 deaths occurred, with 10 attributed to MSO. The 5, 10, and 15-year OS rates were 91.4, 87.7, and 83.5%, respectively. The 5, 10, and 15-year DSS rates were 93.8, 90.0, and 85.7%, respectively. Logistic regression revealed that International Federation of Gynecology and Obstetrics (FIGO) stage IV was the only risk factor for DFS [*p* < 0.001; odds ratio (OR) 7.328; 95% CI 3.103–16.885, FIGO stage IV vs. stage I; *p* = 0.021; OR 4.750, 95% CI 1.264–17.856, FIGO stage IV vs. stage II-III]. The multivariate Cox regression analysis showed that poor differentiation was the only risk factor for both OS (*p* = 0.005, OR 6.406; 95% CI 1.730–23.717) and DSS (*p* = 0.001, OR 9.664; 95% CI 2.409–38.760), while age ≥45 years was the prognostic predictor for OS (*p* = 0.038, OR 4.959; 95% CI 1.093–22.508).

**Conclusion:** Survival outcomes were excellent in patients with MSO, irrespective of the treatment strategy, FIGO stage IV, age ≥45 years, and poor differentiation of tumors were the independent risk factors.

## Introduction

Thyroid tissue can be found in ~15% of the ovarian teratoma. However, thyroid tissue must comprise at least 50% of the overall tissue to be defined as struma ovarii (1, 2). Struma ovarii is rare and accounts for <5% of the ovarian teratoma and about 1% of all the ovarian tumors (3–5). Among them, 5 to 10% are histologically malignant and resemble thyroid carcinoma, and are called MSO (6, 7). Siegel et al. (8) reviewed that there were about 200 documented cases of MSO in the English literature so far. MSO is most commonly seen in women aged between 40 and 50 years but lacks distinct clinical manifestations. Currently, the survival outcomes and prognostic factors of MSO have not been well-defined due to its rarity.

In 2015, Goffredo et al. first reported an excellent survival in 68 cases of MSO, irrespective of the treatment strategies (9). Although their study included patients with and without metastasis, they did not evaluate risk factors for overall survival (OS). Our previous study found a similar OS rate in patients with MSO (confined to the ovary) and metastatic MSO (10, 11). We found that age ≥55 years was a strong risk factor for OS in patients with metastatic MSO but did not find any statistically significant predictive factor for OS in patients with MSO confined to the ovary. Ayhan et al. also investigated the progression-free survival and 5-year OS rate in the patients with MSO in a literature review (12). However, this research did not assess long-term prognosis and risk factors, such as OS at 10 years or longer, as suggested by a previous study that recommended that long-term follow-up should be at least 20 years and the median recurrence interval was 14 years (11). Moreover, these studies did not examine disease-specific survival (DSS). Therefore, the prognostic factors in patients with MSO, including those with or without metastatic MSO at initial diagnosis, remain unclear. Disease-free survival (DFS), OS, and DSS needed to be evaluated in a larger cohort of patients with MSO.

The objective of this study was to assess the survival outcomes in patients with MSO, including the cases where the disease was confined to the ovary and the ones with extra-ovarian metastasis. We also investigated potential predictors for DFS, OS, and DSS in this special population. We comprehensively evaluated a total of 194 cases, including one patient from our hospital and 193 cases documented in the English literature in the last 80 years.

## Methods

### Case Presentation

The Ethics Committee of Peking Union Medical College Hospital approved this retrospective study. A 46-year-old woman visited our hospital on March 27, 2009 with a pelvic mass, discovered during the routine physical examination. Pelvic ultrasonography revealed a heterogenous echo mass in the left posterior position of the uterus with a size of about 7.4 × 3.1 cm. The boundary of the mass was not clear and the shape was irregular. No abnormality of the uterus and the right appendix was detected and ascites were absent. Furthermore, serum tumor markers (CA 125, CA19-9, CA 153, CEA, and AFP) were within the normal range.

**Figure 1:**
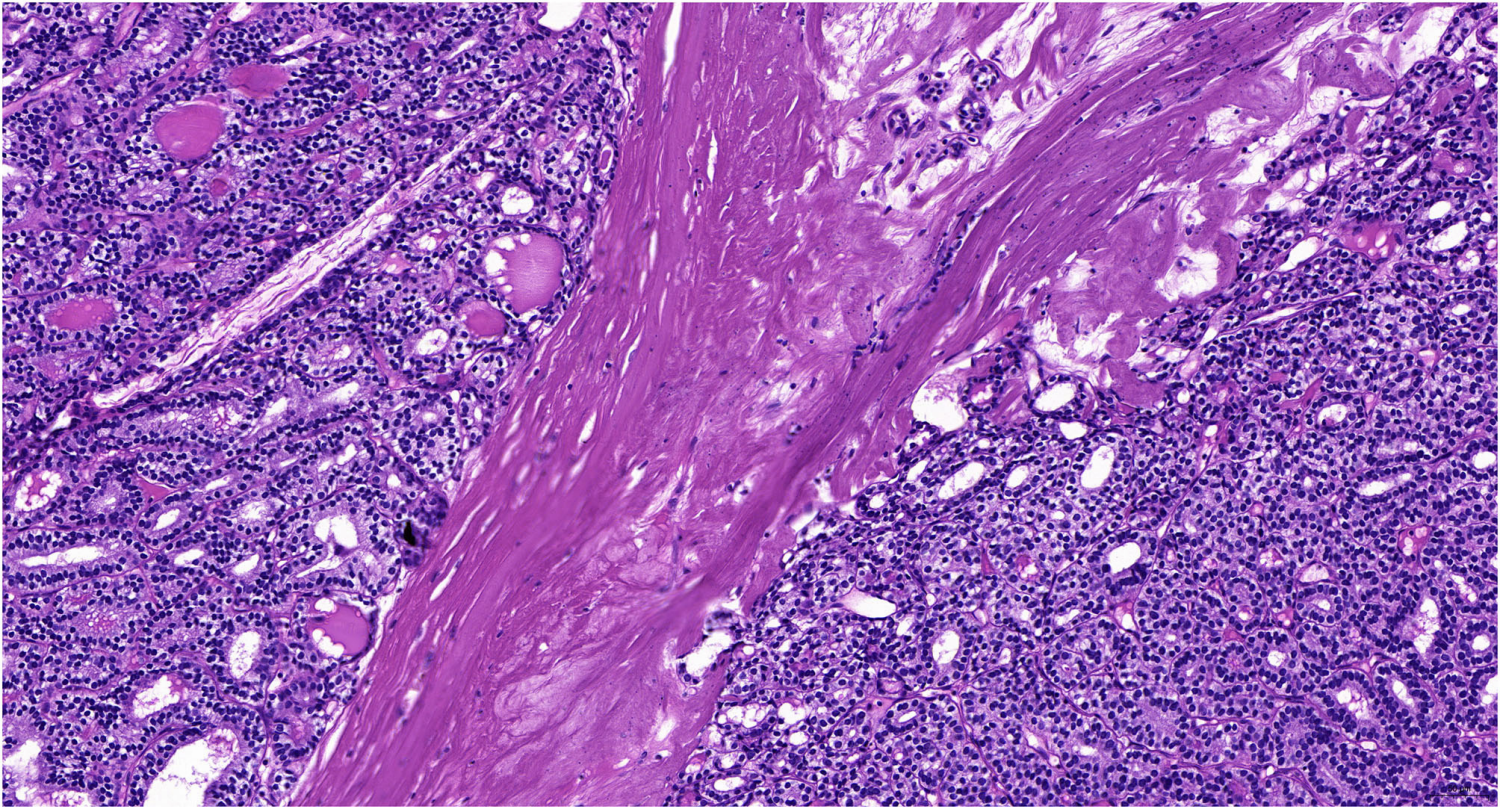
The pathological results of follicular carcinoma arising in the struma ovarii in our case (H&E staining, 400X).

She underwent laparoscopic left unilateral salpingo-oophorectomy (USO) on March 30 and the mass was found in the left ovary without metastasis and ascites. The intraoperative frozen pathological examination revealed ovarian malignant teratoma (MSO). After MSO was diagnosed, she received an optimal debulking surgery, including total abdominal hysterectomy (TAH)/bilateral salpingo-oophorectomy (BSO), omentectomy, and pelvic lymphadenectomy without residual lesion. The definitive paraffin pathological result was follicular thyroid carcinoma with struma ovarii confined to the left ovary (Figure 1). The stage was FIGO stage IA. She underwent four cycles of chemotherapy (cisplatin plus etoposide). She did not receive thyroidectomy and radioiodine therapy (RAI) therapy since subsequent thyroid ultrasonography and thyroid function tests were negative.

The postoperative recovery was uneventful and she was discharged 5 days after surgery. Subsequent follow-up for more than 12 years results revealed no recurrence of MSO and occurrence of primary thyroid cancer.

### Literature Review

We performed a systematic literature review to collect all available cases published in the English language from 1940 to 2021. PubMed, Embase, and Scopus were searched using the following keywords: “malignant struma ovarii,” “metastatic malignant struma ovarii,” “papillary thyroid carcinoma,” “follicular thyroid carcinoma,” “follicular variant of papillary thyroid carcinoma,” “thyroid carcinoma arising in struma ovarii,” and “struma ovarii.” The relevant references cited within these articles were also reviewed. The exclusion criteria were patients with benign struma ovarii, MSO, or metastatic MSO found by autopsy, lack of demographic data, pathological results, treatment methods, or exact follow-up information. The detailed inclusion process is summarized in Supplementary Figure 1. A total of 193 cases of MSO were selected and a database was generated including demographic characteristics, survival outcomes, and clinical, pathological, and treatment features of these 194 cases.

Potential independent variables that might predict disease prognosis were examined, including age at diagnosis (<45, ≥45 years, we set the cut-point as per the American Joint Committee on Cancer (AJCC) staging system for differentiated thyroid cancers and related researches (13, 14)), pathological subtypes (follicular carcinoma or not; well-differentiated or poorly differentiated), tumor size (<8, ≥8 cm, the cut-point was selected as 8 cm after assessing different sizes and was also based on the previous study), surgical options (no surgery, conservative surgery or aggressive surgery), adjuvant therapy (with or without RAI) and FIGO stage (15) (stage I, stage II-III, stage IV). In this study, conservative surgery was defined as ovarian cystectomy with or without metastases resection, USO and bilateral salpingo-oophorectomy (BSO) with or without omentectomy, simple metastases resection, while aggressive surgery was defined as hysterectomy with BSO (H/BSO), or debulking surgery, as described in our previous study (10, 11). DFS was defined as alive with no evidence of disease (NED) after treatment. OS was defined as the time from the date of initial diagnosis to death or final follow-up. DSS was defined as the time from the date of the initial diagnosis to MSO related death or final follow-up.

### Statistical Analysis

Continuous variables that are normally distributed are described by means ± SD (range). Otherwise, they are presented as medians and interquartile ranges. Counts (percentages) are used to express discrete variables. Categorical variables were compared by the chi-squared test. Survival curves were established by the Kaplan–Meier method, and the log-rank test was used to compare survival rates between subgroups. Univariate analysis was performed to assess clinical prognostic factors for DFS, OS, and DSS. Factors with *p* < 0.10 were subjected to multivariate analysis using the logistic regression or Cox regression model to identify independent prognostic factors. A two-tailed *p* < 0.05 was considered significant. We used the SPSS (Version 21.0; SPSS Incorporation, Chicago, Illinois, USA) or the GraphPad Prism (version 8.0) software to conduct statistical analysis.

## Results

A total of 194 patients were included in this retrospective analysis, with a median age of 46.0 years (Supplementary Tables 1A,B). The age at diagnosis varied with 19.6% aged between 18 to 35 years. The most commonly affected women were aged between 40 and 50 years (25.8%), followed by women aged between 30 and 39 years (24.2%), and women aged between 50 and 59 years (19.6%). At the time of diagnosis of MSO, lesions were confined to the ovary in 142 cases (73.2%) while the other 52 cases (26.8%) had extraovarian metastasis.

Papillary carcinoma was the most prevalent pathological subtype (37.1%), followed by the follicular variant of papillary carcinoma, follicular carcinoma, unspecified differentiated thyroid carcinoma, and mixed follicular-papillary carcinoma. A total of 9 cases (4.6%) were diagnosed as poorly differentiated thyroid carcinoma. A total of 87 cases (48.5%) were treated with conservative surgery, followed by aggressive surgery (39.2%), only 4 (2.1%) patients did not receive any surgery. More than half (53.1%) cases did not receive any postoperative adjuvant therapy, 35.6% of them underwent RAI and 8.8% received chemotherapy. Only 5 cases received external beam radiotherapy (Table 1).

**Table 1:** Characteristics and clinical outcomes of patients with MSO.

**Clinical characteristics**	***N* = 194**		***N* = 194**
**Age (y)**		**Time of follow-up (y)**	
Mean 46.7 ± 14.6		Mean 5.21	
Median 46.0 (11–82)		Median 2.75 (0.04–41)	
**Primary TC in thyroid**	12 (6.2%)	**FIGO stage**	
Tumor size (N = 121)		Stage I	142 (73.2%)
8.6 ± 4.7 cm (0.50–25.0)		Stage II	4 (2.1%)
**Pathology**		Stage III	14 (7.2%)
PTC	72 (37.1%)	Stage IV	34 (17.5%)
FVPTC	54 (27.8%)	**Adjuvant therapy**	
FTC (poorly differentiated FTC)	53 (4) (27.3%)	No	103 (53.1%)
unspecified	7 (3.6%)	RAI	65 (33.5%)
Poorly differentiated TC	9 (4.6%)	Chemotherapy	12 (6.2%)
**Surgery**		EBRT/radiotherapy	4 (2.05%)
No	4 (2.1%)	RAI + chemotherapy	4 (2.05%)
Cystectomy with/without metastasectomy	14 (7.2%)	EBRT + chemotherapy	1 (0.5%)
USO with/without metastasectomy	57 (29.4%)	NA	5 (2.6%)
BSO with/without metastasectomy	16 (8.2%)	**Clinical outcomes**	
Hysterectomy + BSO	33 (17.0%)	No evidence of disease	146 (75.3%)
Debulking surgery	43 (22.2%)	Alive with disease	35 (18.0%)
Simple metastectomy	7 (2.6%)	Death related to MSO	10 (5.2%)
NA	20 (10.3%)	Died of other diseases	3 (1.5%)

*MSO, malignant struma ovarii; PTC, papillary thyroid carcinoma; FTC, follicular thyroid carcinoma; FVPTC, follicular variant of papillary thyroid carcinoma; (D)TC, (differentiated) thyroid carcinoma; PDC, poorly differentiated thyroid carcinoma; USO, unilateral salpingo-oophorectomy; BSO, bilateral salpingo-oophorectomy; RAI, radioiodine therapy; EBRT, external beam radiotherapy; NA, not applicable*.

At the final follow-up, 146 patients (75.3%) cases showed NED, 35 (18.0%) cases were alive with disease (AWD), 10 (5.2%) cases died of the disease and 3 (1.5%) died of other diseases (DOD, 1 died of multiple myeloma, 1 died of myocardial infarction, while 1 died of severe chronic obstructive pulmonary disease). The results of the potential risk factors associated with AWD/DOD, identified by univariate and multivariate analyses, were summarized in Supplementary Table 2. The univariate analysis revealed that advanced disease stage and follicular pathology subtypes were associated with AWD/DOD. More aggressive surgical interventions showed a higher possibility of achieving better prognoses. The tumor size was not included since nearly 40% of the reviewed cases did not report exact tumor sizes. The further multivariate logistic regression showed that FIGO stage IV disease [*p* < 0.001; odds ratio (OR) 7.328; 95% CI 3.103–16.885, FIGO stage IV vs. stage I; *p* = 0.021; OR 4.750, 95% CI 1.264–17.856, FIGO stage IV vs. stage II-III] was the only risk factor for AWD/DOD.

**Figure 2:**
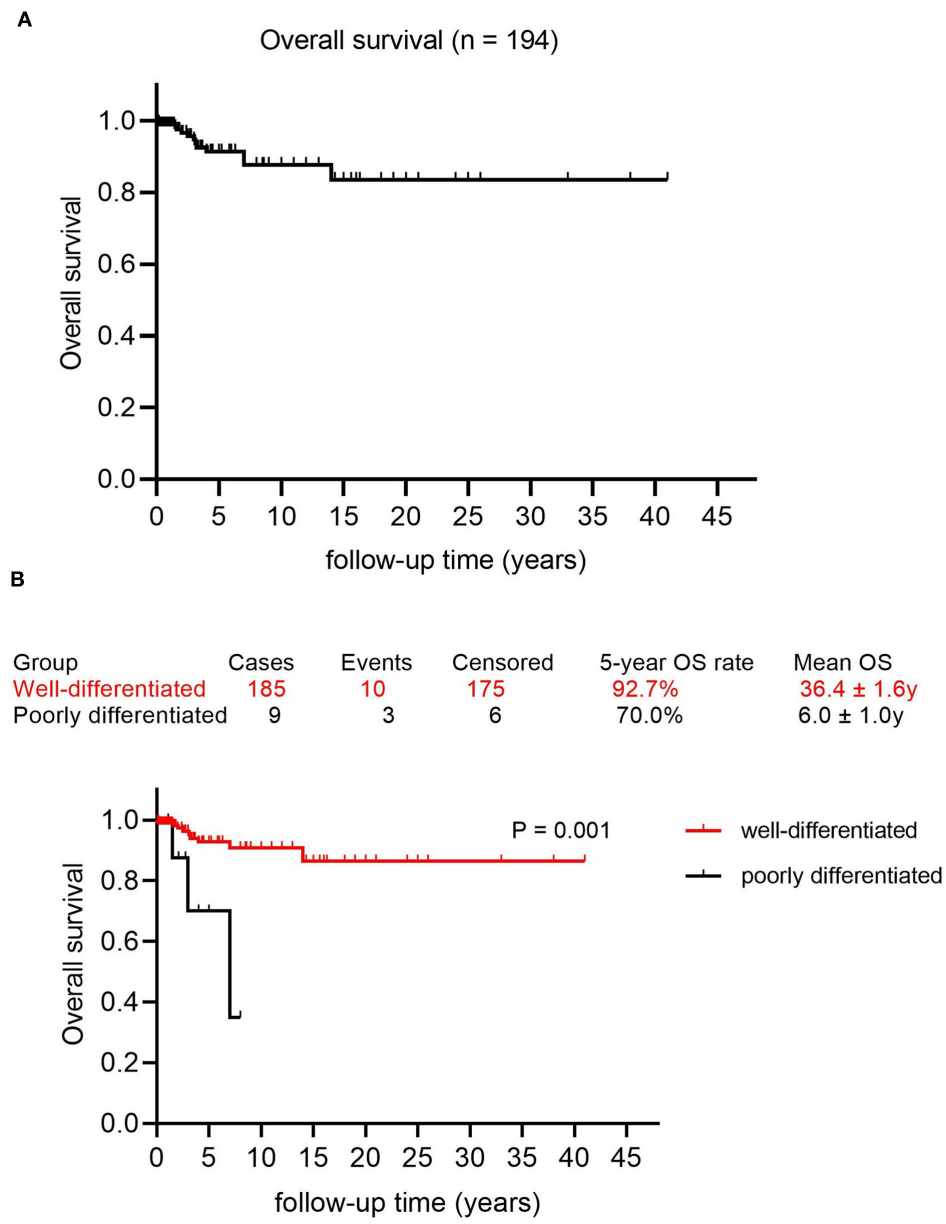
Survival curves in patients with malignant struma ovarii (MSO). **(A)** Overall survival curve in this study. **(B)** The Kaplan–Meier (log-rank) test showed a significantly different OS rate between patients with well-differentiated MSO and poorly differentiated MSO (*p* = 0.001).

**Figure 3:**
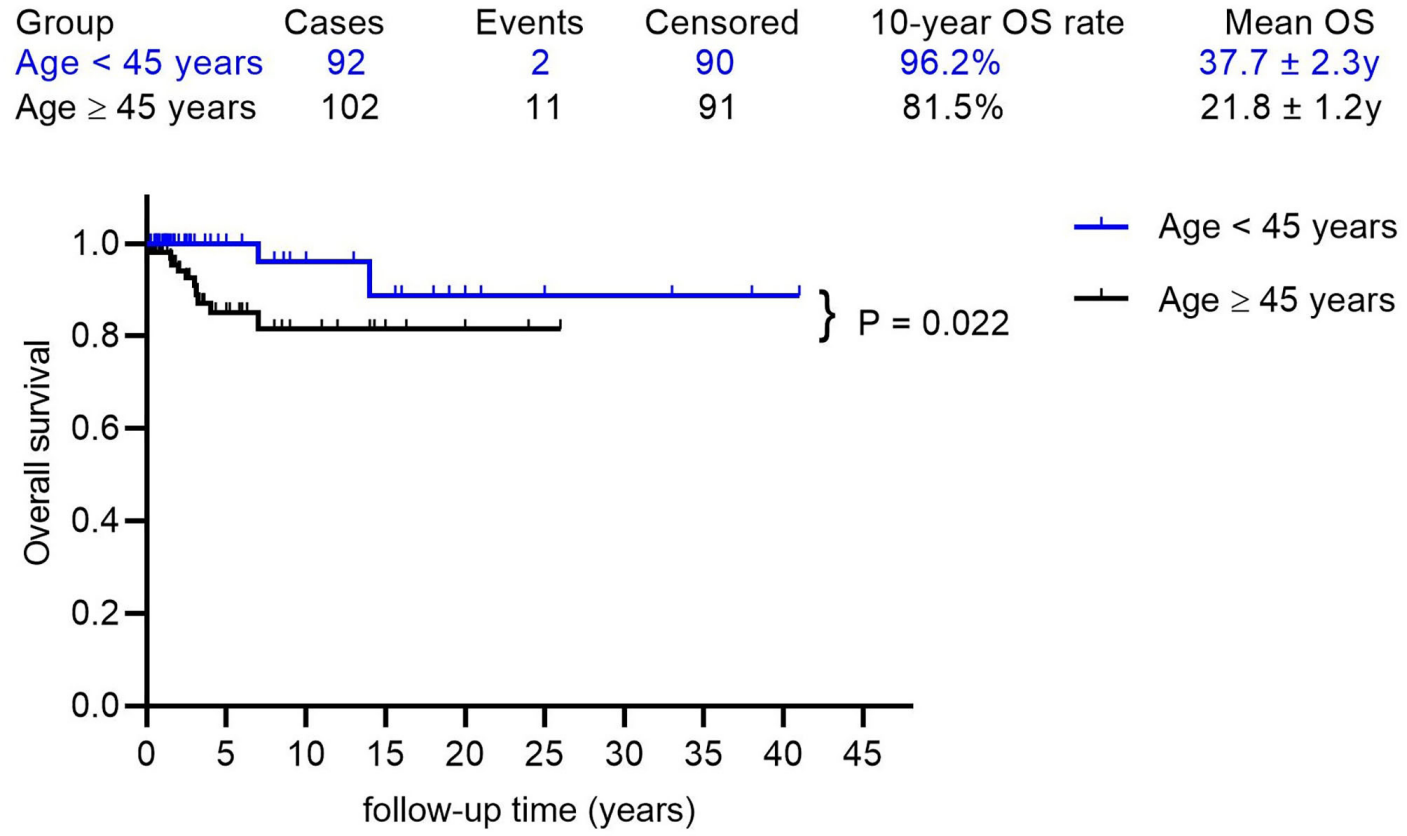
The Kaplan-Meier (log-rank) test showed a significantly different OS rate between patients with ages < 45 years and ages ≥ 45 years (*p* = 0.022).

The mean follow-up time was 5.21 years, with a median of 2.75 years. The 5, 10, and 15 year OS rates were 91.4, 87.7, and 83.5%, respectively, with a mean OS of 35.3 years (95% CI: 32.0–38.6) (Figure 2A). Factors that may affect survival outcomes are summarized in Supplementary Table 3, and univariate analysis showed that age (< 45 years vs. age ≥ 45 years; *p* = 0.022, Figure 3) and poor differentiation (*p* = 0.001, Figure 2B) were significantly associated with disease prognosis. Age and differentiation degree were used for further multivariable analysis, where an age ≥ 45 years (*p* = 0.038, OR 4.959; 95% CI 1.093–22.508) and poor differentiation (*p* = 0.005, OR 6.406; 95% CI 1.730–23.717) remained statistically significant.

**Figure 4:**
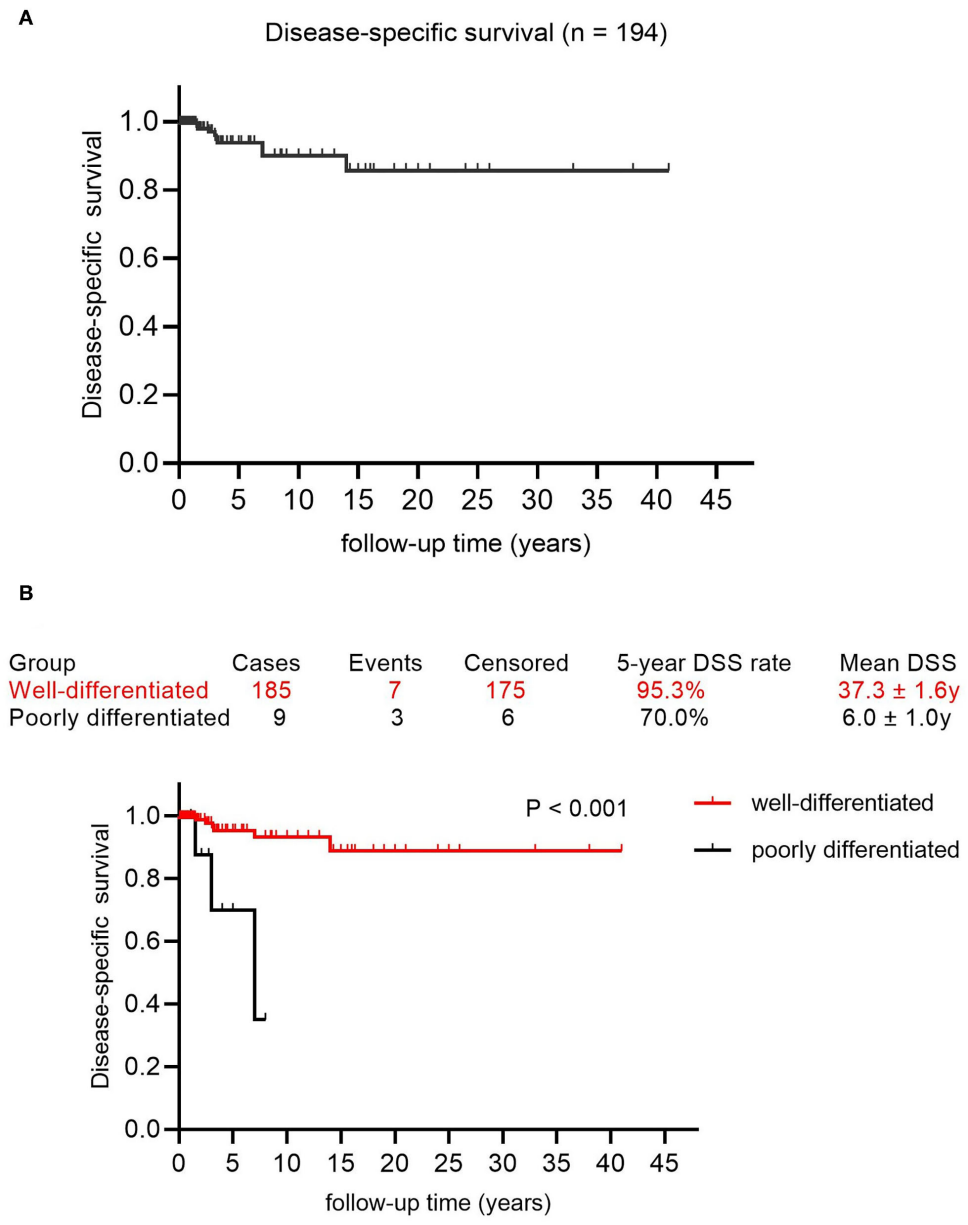
The survival curves in patients with MSO. **(A)** Disease-specific survival in this cohort. **(B)** The Kaplan-Meier (log-rank) test demonstrated the significantly different DSS rate between patients with well-differentiated MSO and poorly differentiated MSO (*p* < 0.001).

Besides, the DSS rates at 5, 10, nd 15 years were 93.8, 90.0, and 85.7%, respectively, with a mean DSS of 36.1 years (95% CI: 32.9–39.4) (Figure 4A). The potential prognostic predictors are summarized in Supplementary Table 4 and univariate analysis showed that poor differentiation (*p* < 0.001, Figure 4B) was significantly associated with adverse prognosis, while age ≥ 45 years and advanced FIGO stage were likely to predict poor prognosis. These three factors were analyzed further using the multivariable Cox regression analysis, whereas the poor differentiation of tumors was the only risk factor for DSS (*p* = 0.001, OR 9.664; 95% CI 2.409–38.760).

## Discussion

This study investigated the prognosis of patients with MSO, including cases confined to the ovary and cases with metastatic MSO, in the largest patient cohort. We evaluated potential risk factors for DFS, OS, and DSS in this population. The prognosis of MSO was remarkable and FIGO stage IV predicted a decreased probability of DFS. Age over 45 years negatively affected OS and poor differentiation of tumors was the only statically significant prognostic factor of both OS and DSS in our study. This study adds new insights into the prognosis of MSO and may help to optimize the management of this disease.

Several large cohort studies showed similar OS outcomes in patients with MSO, regardless of the presence of metastatic lesions at initial diagnosis (9–12). In 2015, a study of 68 patients with MSO showed an OS rate of 96.7, 94.3, and 84.9% in 5, 10, and 20 years, respectively (9). Recently, we evaluated the OS in patients with or without extraovarian spread. The OS rate in patients with metastatic MSO in 5, 10, and 15-year OS rates were 89.3, 82.4, and 65.9%, respectively (10, 11). In patients (*n* = 125) with MSO confined to the ovary, the OS rate was 91.5, 85.2, and 85.2% in 5, 10, and 20 years, respectively. Another systemic review showed the 5-year progress-free survival to be 72.5% (12). Our study had the largest patient cohort and confirmed the excellent prognosis of MSO. Furthermore, our study investigated the DFS and DSS in this population, which may help to make better understand the survival outcomes in patients suffering from this rare disease.

In this study, a novel finding was that poor differentiation of tumors was the only risk factor of OS and DSS. This finding was consistent with previous observations where poorly differentiated carcinomas were associated with aggressive clinical features, such as a higher risk of disease progression, and less sensitivity to RAI therapy (16). The 5-year OS rate in the poorly differentiated thyroid cancer in the neck was 62–85% (17, 18), similar to that 5-year OS rate in our study (70.0%). However, the 5-year OS rate in the differentiated thyroid carcinoma was > 90% (19), comparable with the 5-years OS rate (92.7%) and DSS rate (95.3%) in this study. Moreover, poorly differentiated thyroid carcinoma did not usually respond well to the same modalities of adjuvant treatment compared with differentiated thyroid carcinoma. RAI avidity of poorly differentiated thyroid carcinoma was also inconsistent depending on tumor heterogeneity and variable composed of less well-differentiated tumors (20). Besides, the efficacy of external beam radiation therapy is also controversial and no significant survival improvement has been noted (21). For poorly differentiated thyroid carcinoma, age over 45 years and distant metastasis at presentation were predictors of poor survival outcomes (17, 18). Poorly differentiated thyroid carcinoma show significantly increased tumor burden at the molecular level (22) and is often presented at an older age and advanced stage which are both associated with aggressive biology and loss of RAI avidity (23, 24). Although patients with poorly differentiated MSO only accounted for 4.6% of the total patients, more rigorous follow-up and surveillance must be conducted to improve the prognosis.

Differentiated thyroid cancer is the only malignant tumor that includes age as part of the AJCC staging system (13) and selects it as an important prognostic predictor (14, 16, 25). We also found that age ≥ 45 years predicted an adverse OS. Similarly, age over 55 years (*p* = 0.006; OR: 9.362; 95% CI: 1.895–46.246) was an independent risk factor associated with mortality in a previous study on metastatic MSO (10). The earlier AJCC staging system had a cut-off of 45 years at risk stratifications and it has been changed to 55 years in the latest version (26). The median age of the patients in this study was 46 years, so we decided to set the cut-off as 45 years. The reason behind the poor survival outcomes in the older patients remain unclear. Previous study indicated that older patients were less likely to show an effective treatment response to RAI therapy (27), possibly due to age-dependent variations in the expression of the iodine transporters involved in iodine uptake (28). However, this effect was absent in DSS in this study, which maybe because of the predominant effect of differentiation and the relatively minor influence of age. However, the age-dependent risk on OS should still need to be highlighted in MSO-managing strategy.

The disease stage was strongly associated with survival outcomes in the common epithelial ovarian carcinoma and the FIGO stage as a prognosis predictor has been well established (29). Yassa et al. suggested patients with MSO outside the ovaries should be considered “high risk” (3), but the FIGO stage was not clear in this definition. In 2020, a study demonstrated that the FIGO stage was an independent factor in predicting disease condition following treatment in metastatic MSO (FIGO stage IV vs. stage II-III, *p* = 0.002; OR: 5.333; 95% CI: 1.839–15.471) (10). This study confirmed that FIGO stage IV is a prognostic predictor using a larger sample size. Patients with stage IV disease had a significantly lower probability of achieving DFS compared with those with stage I or stage II-III diseases. This may be because the earlier stage MSO has less tumor burden and more favorable histology, responsive to RAI therapy, or is easily resected by surgery. However, the DFS did not differ between patients with stage I and stage II-III diseases (*P* = 0.497). Patients with or without extraovarian diseases at diagnosis (FIGO stage I vs. stage II-IV) showed no statistically significant difference in both OS (*p* = 0.294) and DSS (*p* = 0.077). Moreover, the FIGO stage showed no statistically significant effect on OS and DSS in the multivariable Cox regression analysis, which may attribute to the high efficacy of adjuvant therapy especially RAI therapy (10, 11). Most of the patients achieved NED or AWD and the risk of death related to MSO was quite low. Our study shows novel insight into the impact of disease stage on survival outcomes.

Other factors that may affect the survival outcomes of patients with MSO had also been assessed, including surgical options, tumor size, follicular pathologic subtype, and RAI therapy, but none of them was significant in the multivariate regression analysis. These results were also consistent with the previous 2 studies (10, 11). Although Robboy et al. (30) and Shaco-Levy et al. (31) proposed tumor size or stromal size/component as risk factors for disease recurrence, disease progression, and death, subsequent study showed the clinical outcome of MSO did not correlate with microscopic morphology. No specific histologic features affected disease outcomes (32). We found that there were no reports of tumor size in about 40% of patients. Previous research indicated that it was extremely difficult to measure tumor size where cancer and teratomatous components blended together (10). Moreover, the presence of ascites, capsular involvement, and lymphovascular invasion were seldom detailed in the literature, making it challenging to link these factors with disease prognosis even though some were risk factors in primary thyroid cancer (33). However, since the actual median follow-up time in our study was relatively short, and the previous studies suggested long-term follow-up of at least 20 years (1), the accurate true DFS, OS, and DSS and their risk factors are still needed to be further studied.

We proposed a risk stratification method and management based on our results. Age ≥45 years can be used as a risk factor of OS in patients with MSO, including those with or without metastatic diseases, while age ≥55 years predicted significantly poor survival outcomes in patients with metastatic MSO. Poor differentiation was an important prognostic factor associated with adverse OS and DSS in this population, while the FIGO stage indicated the disease condition after treatment. Patients with MSO confined to the ovary were potentially considered “low risk” because no identified risk factor was verified, which is similar to risk stratification of thyroid cancer in the neck (33). Conservative surgery is recommended in this population to maintain long-term quality of life, regardless the presence of metastatic lesions or not. However, the choice of specific surgical options should be based on the published study (10, 11). RAI should be recommended to patients with metastatic MSO, while in patients without extraovarian spreading lesion, RAI may be used to improve prognosis but it may be a potential risk in impairing ovarian function (10, 11, 34–36). However, validation of this risk stratification and relative management strategy needs to be further assessed.

Several limitations of this study must be noted. The intrinsic heterogeneity of this study must be mentioned since most cases are reviewed from the literature due to the rarity of the disease. Intrahospital and intraphysician differences of the same surgical option may affect the prognosis of patients. Besides, dozens of patients with MSO who had prognosis data but lacked exact follow-up details were excluded from analysis, which may have biased the accurate impact of identified risk factors on survival outcomes. Furthermore, the type of schedule of chemotherapy and the type of drugs administered varied in patients, which may also impact the survival outcomes and make it more challenging to evaluate the role of chemotherapy in patients with MSO. Due to its rarity, more research is urgently needed to verify our results.

## Conclusion

Survival outcomes were excellent in patients with MSO irrespective of the treatment strategy. FIGO stage IV, age ≥ 45 years, and poor differentiation of the tumors were the independent risk factors of prognosis in patients with MSO.

## Data Availability Statement

The original contributions presented in the study are included in the article/Supplementary Material, further inquiries can be directed to the corresponding author/s.

## Ethics Statement

This retrospective study was approved by the Ethics Committee of Peking Union Medical College Hospital (Reference Number: S-K1198).

## Author Contributions

SL conducted the statistical analysis, wrote the manuscript, and participated in the study design. SK completed the surgery and treatment strategies. XW, MY, and XZ participated in the literature review and completed the work of follow-up. JY conceived and designed the study. All authors have read and approved the final version of the manuscript.

## Funding

This study was funded by Chinese Academy of Medical Sciences (CAMS) Innovation Fund for Medical Sciences (CIFMS) (CIFMS-2017-I2M-1-002) and National High Technology Research and Development Program of China (2012AA02A507).

## Conflict of Interest

The authors declare that the research was conducted in the absence of any commercial or financial relationships that could be construed as a potential conflict of interest.

## Publisher's Note

All claims expressed in this article are solely those of the authors and do not necessarily represent those of their affiliated organizations, or those of the publisher, the editors and the reviewers. Any product that may be evaluated in this article, or claim that may be made by its manufacturer, is not guaranteed or endorsed by the publisher.

## Supplementary Material

Supplementary Figure 1 The detailed inclusion process is according to the PRISMA flow diagram in this study.

Supplementary Table 1 Database of our study **(A)** patients with malignant struma ovarii (MSO) confined to the ovary; **(B)** patients with metastatic MSO.

Supplementary Table 2 The univariate and multivariate analysis of clinical outcomes.

Supplementary Table 3 The univariate and multivariate analysis of overall survival (OS).

Supplementary Table 4 The univariate and multivariate analysis of disease-specific survival (DSS).
