# Supplementary figures and images for: Survival Outcomes and Prognostic Predictors in Patients With Malignant Struma Ovarii
Source: Front Med (Lausanne). 2021 Dec 23;8:774691. doi: 10.3389/fmed.2021.774691 (PMC8733601; doi:10.3389/fmed.2021.774691)

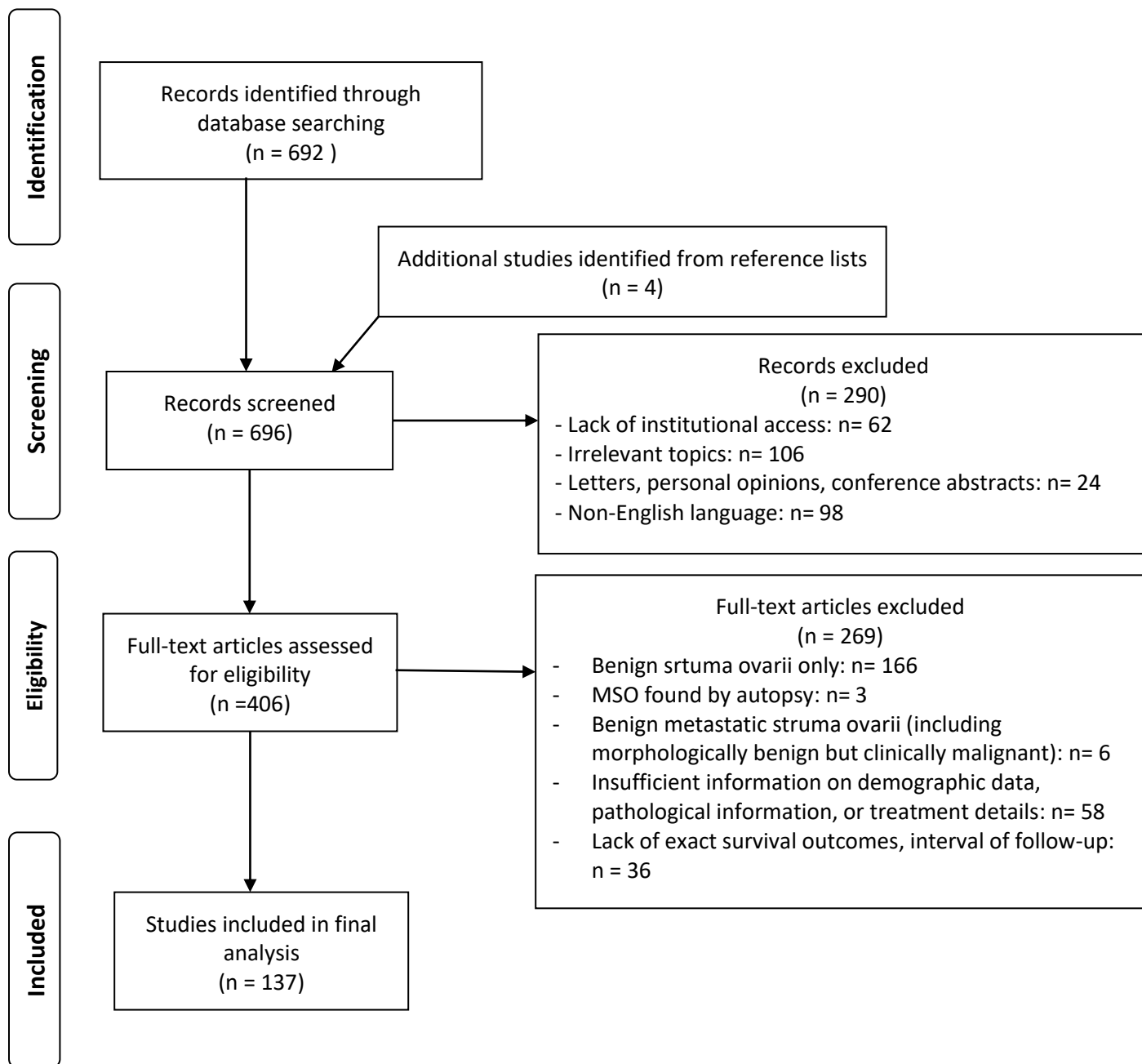

Supplement: Supplementary Figure 1 — The detailed inclusion process is according to the PRISMA flow diagram in this study. [file Data_Sheet_1.PDF]
