# Supplementary material for: Survival Outcomes and Prognostic Predictors in Patients With Malignant Struma Ovarii
Source: Front Med (Lausanne). 2021 Dec 23;8:774691. doi: 10.3389/fmed.2021.774691 (PMC8733601; doi:10.3389/fmed.2021.774691)
Supplement: Supplementary file 2 [file Table_1.DOCX]

Table S1a. Database of our study (patients with MSO confined to the ovary).

| Reference | No. | Age  (y) | Pathology;  Mass size | Surgery | Adjuvant  therapy | R | RFS | Recurrence  site | Surgery at recurrence | Adjuvant  In R | Results of follow-up |
| --- | --- | --- | --- | --- | --- | --- | --- | --- | --- | --- | --- |
| Woodruff &  Markley  (1957) | 1 | 26 | FTC;  7cm | Ovarian cystectomy | N | Y | 5y | Lungs,  Anterior superior mediastinum | N | TT RAI*2(350mc;100mc) | AWD at 8y |
| Kempers  (1970) | 2 | 42 | FTC;  18cm | TAH + BSO | N | Y | 16y | Peritoneum; diaphragm; iliac nodes; lungs | debulking surgery | TT RAI*2 | NED at 21y |
|  | 3 | 46 | FTC; >6CM | TAH + BSO | N | Y | 7y | Liver; bone | N | N | DOD at 7y |
|  | 4 | 54 | Mixed FTC and PTC; >6cm | TAH + BSO | N | N | 15y | N | N | N | NED at 15y |
|  | 5 | 54 | PTC; >6cm | (USO) RSO | TT | N | 26y | N | N | N | NED at 26y |
| Graff (1980) | 6 | 72 | FTC; 7cm | TAH + BSO + omentectomy (Debulking) | N | N | 3y | N | N | N | NED at 3y |
|  | 7 | 30 | Poorly-differ  Mixed FTC+PTC;  13cm | USO(LSO) | N | N | 33m | N | N | N | NED at 33m |
| O’ Connell  (1990) | 8 | 35 | PTC; mixed PTC+ FTC at R | USO(LSO) + cystectomy | N | Y | 11y | Contralateral ovary; peritoneum | TAH + RSO + omentectomy  (debulking) | RAI*1;  50mCi | NED at 38y |
|  | 9 | 35 | FTC;  15cm | TAH + BSO | N | N | 6m | N | N | N | NED at 6m |
| Brunskill  (1990) | 10 | 38 | FVPTC;  8*6cm | TAH + BSO + omentectomy  (debulking) | N | N | 2y | N | N | N | NED at 2y |
| Ayhan  (1993) | 11 | 66 | FTC;  12*8cm | USO + omentectomy | Chemotherapy | Y | 5y | ileocecal  serosa | TAH + USO+ omentectomy + LN  (debulking) | Multi-Chemo-therapy | NED at 8y |
|  | 12 | 37 | PTC;  9*6cm | USO; TAH + USO+  Omentectomy + LN (debulking) | N | N | 10y | N | N | N | NED at 10y |
| Piura  (1995) | 13 | 65 | FTC;  13cm | TAH + BSO | N | N | 70m | N | N | N | NED at 70m |
| Brenner  (1996) | 14 | 49 | FTC;  5cm | USO(LSO) | TT, RAI*2  3,6GBq | Y | 0.8y | Urinary bladder | N | RAI*1  6GBq | NED at 30m |
| Berghella  (1997) | 15 | 32 | PTC;  5.5*3*2cm | Cystectomy;  USO(LSO) + omentectomy | N | N | 2y | N | N | N | NED at 2y |
| Doldi  (1998) | 16 | 58 | PTC;  4cm | Cystectomy | N | N | 1y | N | N | N | NED at 1y |
| Rose  (1998) | 17 | 42 | Mixed PTC + FVPTC;  8*8cm | TAH + LSO + LN;  (debulking) | N | Y | 6m | WBS (-);  TG elevated | N | RAI*2;  29.6mCi;  27.5mCi | NED at 1y |
| Dardik  (1999) | 18 | 28 | FVPTC;  11*8*6cm | Right  Ovariectomy  (USO) | N | Y | 2y | Fallopian tubes;  Paraaortic LN | Celiotomy + TAH + LSO + RS +omentectomy + paraaortic LN  (Debulking) | TT, RAI | NED at 4y |
|  | 19 | 39 | FTC;  PTC in thyroid | (USO)  RSO | N | Y | 0.6y | Hernia sac;  Peritoneum;  omentum | TAH + LSO + omentectomy + tumor resection  (debulking) | TT, RAI,  (150mCi); EBRT (5040 cGy) | NED at 6y |
| Bhansali  (1999) | 20 | 47 | FTC;  7*6cm | TAH + BSO | EBRT;  4500 rads | N | 4y | N | N | N | NED at 4y |
| Matsuda  (2001) | 21 | 48 | PTC | TAH + BSO + omentectomy + LN (debulking) | N | N | 3y | N | N | N | NED at 3y |
| Hemli  (2001) | 22 | 18 | PTC;  7*6*5cm | Cystectomy | N | N | 21m | N | N | N | NED at 21m |
| Kabukcuglu  (2002) | 23 | 52 | FVPTC;  6*5*4cm | TAH + BSO + omentectomy + appendectomy + LN (debulking) | N | N | 2y | N | N | N | NED at 2y |
| Sussman  (2002) | 24 | 53 | FVPTC;  7.5cm | TAH + BSO | N | N | 3m | N | N | N | Died of myocardial infarction at 3m |
| DeSimone  (2003) | 25 | 32 | FTC;  7*5.5*4.8cm | TAH+ BSO | TT, RAI | N | 14m | N | N | N | NED at 14m |
| Volpi  (2003) | 26 | 49 | PTC;  6cm | Staging (BSO + omentectomy + LN) | N | N | 1y | N | N | N | NED at 1y |
| Zannoni  (2004) | 27 | 66 | FVPTC;  12*9cm | TAH + BSO + omentectomy + LN (debulking) | N | N | 6m | N | N | N | NED at 6m |
| Makani  (2004) | 28 | 46 | PTC;  16cm | TAH + BSO | N | N | 32m | N | N | N | NED at 32m |
| Ihalagama  (2004) | 29 | 27 | FTC;  10cm | USO  (RSO) | N | N | 32m | N;  G2P2 later | N | N | NED at 32m |
| Garcia  (2005) | 30 | 22 | FTC;  5*4cm | Right ovariectomy (USO);  Omentectomy + LN + biopsies | N | N | 6y | N | N | N | NED at 6y |
| Bolat  (2005) | 31 | 34 | PTC;  4cm | (USO)RSO + wedge biopsy + LN + omentectomy | N | N | 7m | N | N | N | NED at 7m |
| Zekri (2006) | 32 | 26 | FTC; 12cm | USO | N | Y | 10y | Bone (Skull),  lung | N | TT, RAI*3 (5421, 5455,7400MBq) | AWD at 16y |
| Doganay (2007) | 33 | 53 | PTC; 5cm | TAH + BSO | TT | N | 1y | N | N | N | NED at 1y |
| Schimdt (2007) | 34 | 32 | FVPTC; multiple modules 0.25-6.5cm | NA | NA | N | 20m | N | N | N | NED at 20m |
|  | 35 | 53 | FVPTC; 0.3cm | NA | NA | N | 7m | N | N | N | NED at 7m |
|  | 36 | 52 | FVPTC; 3cm | NA | NA | N | 52m | N | N | N | NED at 52m |
|  | 37 | 42 | FVPTC; Microscopic focus | NA | NA | N | 33m | N | N | N | NED at 33m |
|  | 38 | 27 | PTC | NA | NA | Y | 22y; 41y | Retroperitoneum | NA | NA | AWD at 41y |
| Tomee (2008) | 39 | 51 | PTC; 10cm | USO | N | N | 4y | N | N | N | Die of COPD at 4y |
| Yassa (2008) | 40 | 25 | PTC; 10cm | USO | thyroxine | N | 1y | N | N | N | NED at 1y |
| Janszen (2008) | 41 | 33 | FVPTC; 10*15cm | TAH + BSO | N | Y | 3y | Liver, lymph nodes | N | TT, RAI*3(5550,1850,5550MBq) | AWD at 8y |
|  | 42 | 52 | FTC, 10*8cm; PTC in cervical thyroid | BSO | TT; RAI (5550MBq) | N | 2y | N | N | N | NED at 2y |
|  | 43 | 32 | PTC;8*4cm | USO | TT, RAI(5550MBq) | N | 6m | N | N | N | NED at 6m |
| Prasad (2008) | 44 | 40 | FTC; 10cm | TAH + BSO, omentectomy (debulking) | TT, thyroxin | N | 4y | N | N | N | NED at 4y |
| Roth  （2008） | 45 | 34 | PTC;  17*14*7.5cm | Right oophorectomy + left cystectomy;  (USO) | N | N | 25y | N | N | N | NED at 25y |
| Garg (2009) | 46 | 38 | FVPTC; 8mm | ovarian cystectomy | N | Y | 3y | Contralateral ovary,  Peritoneum (cul-de-sac);  omentum | USO, biopsies of left ovary, omentum, and  peritoneum | TT, RAI | AWD at 9y |
|  | 47 | 34 | FVPTC; multiple small  foci | ovarian cystectomy | N | Y | 4y | Uterine serosa, peritoneum,  diaphragm, and liver | TAH + BSO, peritoneal biopsies, diaphragm  tumor excision, liver right lobe resection | TT, RAI | AWD at 10y |
|  | 48 | 32 | PTC; 7mm | LSO and staging | N | N | 14y | N | N | N | NED at 14y |
|  | 49 | 40 | PDC  (poorly-differentiated); 2.5cm | Staging | N | N | 1.1y | N | N | N | NED at 1.1y |
|  | 50 | 42 | PTC; 1.1mm | TAH + BSO; staging | N | N | 1y | N | N | N | NED at 1y |
|  | 51 | 47 | PDC; 5cm | TAH + BSO | N | N | 5y | N | N | N | NED at 5y |
|  | 52 | 51 | FVPTC; 6mm | TAH + BSO | N | N | 4y | N | N | N | NED at 4y |
|  | 53 | 56 | FVPTC; 5mm | TAH + BSO | N | N | 1.3y | N | N | N | NED at 1.3y |
| Wong (2009) | 54 | 44 | FVPTC | USO | TT, RAI (108mCi) | N | 0.5y | N | N | N | NED at 0.5y |
| Yucesoy  (2010) | 55 | 40 | PTC;  15*20cm | TAH + BSO + LN  (debulking) | N | N | 18m | N | N | N | NED at 18m |
| Shaco-levy  (2010) | 56 | 57 | PTC;  15cm | TAH + BSO | N | Y | 11m | Peritoneum, viscera, diaphragm, liver, bowels | N | Chemo-  therapy | DOD at 19m; |
|  | 57 | 54 | PTC;  14cm | TAH + BSO + omentectomy;  (debulking) | N | Y | 2.8y | Local peritoneum | N | N | DOD at 3.2y |
| Marcy  (2010) | 58 | 45 | FVPTC | USO(LSO) | N | Y | 1y | Liver, peritoneum, adrenal, lung, bone(ilium) | TAH + RSO + omentectomy + LN  (ascites +) | TT, RAI*3  (7.4/5.4/6 GBq);  Chemo*4  (C + ADM) | DOD at 37m |
| Sibio  (2010) | 59 | 74 | PTC;  10cm | TAH + BSO +peritoneal benign SO resection + LN  (debulking) | N | N | 7y | N | N | N | NED at 7y |
| Ruel  (2010) | 60 | 42 | FVPTC  (1cm);  PTC at R | Left ovarian cystectomy | N | Y | 5y/15y | Ovary at R1; Lung, bone (rib), para-aortic LN at R2 | (USO)LSO at R1; metastases resection at R2 | TT, RAI*3  (3.9/3.8/  4.9GBq) | NED at 15.6y |
| Yang  (2010) | 61 | 74 | PTC;  13*10cm | TAH + BSO | N | Y | 31m | Colon  (bowel) | LN + tumor resection | TT, RAI | NED at 43m |
| Agrawal  (2010) | 62 | 36 | PTC;  13*11*10cm | USO(RSO) | N | N | 2y | N | N | N | NED at 2y |
| Salman  (2010) | 63 | 67 | PTC;  10*7cm | TAH + BSO +  omental biopsy | N | N | 2y | N | N | N | NED at 2y |
| Kraemer  (2011) | 64 | 40 | FVPTC;  7cm | USO  (RSO) | N | N | 1y | N | N | N | NED at 1y. |
| Tanaka  (2011) | 65 | 50 | FVPTC;  8cm | TAH + LSO | N | N | 14m | N | N | N | NED at 14m |
| Menon  (2011) | 66 | 46 | FVPTC;  14*10cm | TAH + BSO | N | N | 3m | N | N | N | NED at 3m |
| Meringolo  (2011) | 67 | 62 | PTC;  17cm | USO | N | N | 1y | N | N | N | NED at 1y |
| Hinshaw  (2012) | 68 | 74 | FVPTC;  8.5*7.3cm | TAH + BSO + omentectomy + LN (debulking) | N | N | 6m | N | N | N | NED at 6m |
| Jean  (2012) | 69 | 60 | PTC;  6*5cm | BSO + multi biopsies + LN | TT, RAI | N | 2y | N | N | N | NED at 2y |
| Selvaggi  (2012) | 70 | 50 | FTC;  6*5cm | TAH + BSO + omentectomy + LN (debulking);  gastrectomy | N | N | 1y | N | N | N | NED at 1y; coexisted Gastric cavernous angioma |
| kumar  (2012) | 71 | 35 | FVPTC;  10mm | USO(LSO);  Right cystectomy  For teratoma | N | N | 1y | N | N | N | NED at 1y;  Plan to aspiration  Of the thyroid |
| Barrera  (2012) | 72 | 41 | PTC;  10*7*6.5cm | TAH + BSO | TT, RAI*1  (100mCi) | N | 6m | N | N | N | NED at 6m |
| Shrimali  (2012) | 73 | 52 | PTC | Unilateral  Oophorectomy  (USO) | TT, RAI | N | 75m | N | N | N | NED at 75m |
|  | 74 | 53 | FTC | TAH + BSO + omental biopsy | TT, RAI | N | 60m | N | N | N | NED at 60m |
|  | 75 | 50 | PTC | TAH + BSO | TT, RAI | N | 62m | N | N | N | NED at 62m |
|  | 76 | 35 | PTC | USO(LSO) | TT, RAI | N | 17m | N | N | N | NED at 17m |
| Lee  (2012) | 77 | 35 | FTC | Ovarian cystectomy | N | Y | 2y | Liver, peritoneum, diaphragm, right salpinx | Debulking | TT, RAI | AWD at 2.5y |
| Marti  (2012) | 78 | 43 | FTC;  4m | Ovarian cystectomy | N | N | 13y | N | N | N | NED at 13y |
|  | 79 | 44 | PTC;  5cm | BSO + appendectomy | N | N | 9y | N | N | N | NED at 9y |
|  | 80 | 57 | PTC;  4cm | TAH + BSO | N | N | 9y | N | N | N | NED at 9y |
|  | 81 | 44 | PTC;  5cm | TAH + BSO + omentectomy + LN  (debulking) | TT, RAI*2;  29, 150mCi | N | 0.6y | N | N | N | NED at 0.6y;  Synchronous PTC in  Neck (5mm). |
| Collins  (2012) | 82 | 61 | PTC | sTAH + BSO | Chemotherapy | Y | 30m | peritoneum | Tumor resection | TT, RAI | NED at 5y |
| Steinman  (2013) | 83 | 35 | FVPTC | USO(RSO) + LN + omentum/peritoneal  biopsy | N | Y | 11y | Left anterior  Pelvis bone | Tumor resection | TT, RAI*3;  226/  196.2mCi | NED at 14y |
| Leite  (2013) | 84 | 78 | PTC;  10*9cm;  PTC in neck | USO(RSO) | TT | N | 2y | N | N | N | Died of multiple  Myeloma at 2y.  Previous NED |
| Krishna-  Murthy  (2013) | 85 | 51 | FVPTC;  12*8cm;  FVPTC in  neck | TAH + BSO + omentectomy + LN +peritoneal sampling  (debulking) | TT for primary thyroid cancer | N | 6m | N | N | N | NED at 6m. |
| Leong  (2013) | 86 | 42 | PTC; 13.5cm; PTC in thyroid | TAH + BSO | TT, RAI*1;  150mCi | N | 1y | N | N | N | NED at 1y |
| Mardi  (2013) | 87 | 40 | PTC;  11*10*7cm | Ovarian cystectomy | N | N | 6m | N | N | N | NED at 6m |
| Karagko  -unis  (2014) | 88 | 30 | PTC;  5cm | USO(RSO) + omentectomy + LN | TT, RAI*1;  90mCi | N | 9m | N | N | N | NED at 9m |
| Yan  (2014) | 89 | 38 | PTC | USO(RSO) | N | Y | 2y | Liver, retroperitoneal  Lymph nodes | Metastases resection | TT, RAI*3, rh-TSH;  50/402/  355mCi | AWD at 6y |
| Brusca  (2014) | 90 | 30 | FVPTV;  7*3cm | USO(RSO) + appendectomy + peritoneal biopsy | TT, RAI*1;  30mCi | N | 6m | N | N | N | NED at 6m;  PTC in neck (2mm) |
| Park  (2015) | 91 | 80 | FTC;  20cm | Tumor resection;  (cystectomy) | N | N | 20m | N | N | N | NED at 20m |
| Srbovan  （2015） | 92 | 62 | PTC;  1.3mm | TAH + BSO | TT, RAI;  3.7GBq | N | 5m | N | N | N | NED at 5m |
| Tan  (2015) | 93 | 55 | FVPTC | NA | N | N | 11m | N | N | N | NED at 11m |
|  | 94 | 22 | PTC | NA | N | N | 16m | N | N | N | NED at 16m |
|  | 95 | 48 | PTC | NA | N | N | 8.5y | N | N | N | NED at 8.5y |
| Cong  (2015) | 96 | 38 | FTC | Left ovariectomy  (USO) | N | Y | 17y | lung | N | TT, RAI*3;  5.55*3GBq | AWD at 20y |
| Khunamor-  Npong  (2015) | 97 | 21 | Poorly differentiated TC;  15cm | USO(RSO) + LN, omental biopsies + appendectomy | N | Y | 15m | Lung, spinal cord | N | TT, RAI, chemotherapy | DOD at 7y |
| Monti  (2015) | 98 | 41 | FVPTC | Left oophorectomy;  (USO) | TT, RAI | N | 2y | N | N | N | NED at 2y |
| Wei  (2015) | 99 | 72 | FVPTC;  7cm |  | N | N | 3y | N | N | N | NED at 3y |
|  | 100 | 60 | FVPTC;  8.5cm |  | N | N | 2y | N | N | N | NED at 2y |
|  | 101 | 59 | FVPTC;  15cm |  | N | N | 11y | N | N | N | NED at 11y |
|  | 102 | 70 | PTC;  8.9cm |  | N | N | 15y | N | N | N | NED at 15y |
|  | 103 | 60 | FVPTC;  4.6cm |  | TT, RAI | N | 5y | N | N | N | NED at 5y |
|  | 104 | 48 | PTC |  | N | N | 8y | N | N | N | NED at 8y |
|  | 105 | 67 | FVPTC;  0.5cm |  | N | N | 2m | N | N | N | NED at 2m |
|  | 106 | 39 | FVPTC;  4.2cm |  | N | N | 15m | N | N | N | NED at 15m |
|  | 107 | 55 | FVPTC;  7cm |  | N | N | 1m | N | N | N | NED at 1m |
| Fukunaga (2016) | 108 | 66 | Anaplastic carcinoma;  12*10*7.5cm | TAH + BSO | Chemotherapy | N | AWD | N | N | N | AWD at 25m |
| Lara (2016) | 109 | 36 | PTC;  5*4*3cm | USO + omentectomy | TT, RAI (75mCi) | N | 1y | N | N | N | AWD at 1y |
| Oudoux (2016) | 110 | 49 | FVPTC; 7*5.5*3cm | TAH + BSO + omentectomy + LN (Debulking) | TT, RAI  (3.7GBq) | N | 3y | N | N | N | NED at 3y |
| Ma (2016) | 111 | 47 | FVPTC;  5.2cm;  FVPTC in neck | TAH + BSO | TT, RAI | N | 3y | N | N | N | NED at 3y |
| Llueca (2017) | 112 | 43 | PTC; 7cm | USO | N | N | 4y | N | N | N | NED at 4y |
| Middelbeek (2017) | 113 | 55 | PTC; 15cm;  FVPTC in neck | BSO | TT, RAI  (150mCi, performed before BSO) | N | 5y | N | N | N | NED at 5y |
| Gomes-Lima, (2018) | 114 | 67 | PTC, 7.2cm; PTC (cervical thyroid) | BSO | TT, RAI (147.7mCi) | N | 5y | N | N | N | NED at 5y |
| Alamdari (2018) | 115 | 11 | FTC&FVPTC; 11*11*10cm | right  oophorectomy  (USO) | TT, RAI*2(150*2mCi) | N | 8m | N | N | N | NED at 8m |
| Yasutake (2018) | 116 | 66 | PTC; 2.5cm | TAH + BSO + partial omentectomy  (debulking) | N | N | 3m | N | N | N | NED at 3m |
| Wu  (2018) | 117 | 48 | PTC;  10*8cm | Left ovariectomy  (USO) | Chemotherapy | Y | 14y | Colon, rectum, liver, spleen, peritoneum | N | TT, RAI*1;  150mCi  (5.55GBq) | AWD at 14.3y |
| Tzelepis (2019) | 118 | 32 | FVPTC, 6cm; PTC (cervical thyroid) | (USO)LSO, right ovarian cystectomy, partial omentectomy | TT | Y | 5m | mesenteric  lymph nodes | N | RAI  (184.3 mCi) | NED at 1.5y |
| Richa (2019) | 119 | 31 | FTC;  6.5cm | USO (RSO) | RAI | N | 0.5y | N | N | N | NED at 0.5y |
| Hassan (2019) | 120 | 51 | PTC;  8.1*6.7cm | TAH + BSO + omentectomy + appendectomy + pelvic lymphadectomy | N | N | 2y | N | N | N | NED at 2y |
| Khatchap-  Uridze  (2020) | 121 | 50 | PTC;  8.5*8cm | TAH + BSO + omentectomy;  (debulking) | TT, RAI*2;  100/10mCi | N | 3.8y | N | N | N | NED at 45m |
| Gonet  (2020) | 122 | 17 | FVPTC;  17cm | LSO + right ovariectomy;  (BSO) | TT, RAI | N | 4y | N | N | N | NED at 4y |
| Poli (2020) | 123 | 52 | PTC;  1cm | TAH +BSO | N | N | 4y | N | N | N | NED at 4y |
|  | 124 | 66 | FVPTC;  25cm | BSO | N | N | 6y | N | N | N | NED at 6y |
|  | 125 | 63 | PDC;  9cm | BSO | RAI | N | 8y | N | N | N | NED at 8y |
|  | 126 | 66 | FVPTC;  5cm | BSO | N | N | 12y | N | N | N | NED at 12y |
|  | 127 | 56 | PTC;  3cm | BSO | N | N | 20y | N | N | N | NED at 20y |
|  | 128 | 77 | FVPTC;  11cm | BSO | N | N | 2y | N | N | N | NED at 2y |
| Limaiem (2020) | 129 | 75 | FTC;  14*13*8cm | USO (unilateral right adnexectomy) | N | N | 0.5y | N | N | N | NED at 0.5y |
| Li (2020) | 130 | 39 | FTC | USO | N | Y | 9y | lung | N | TT, RAI | AWD at 19y |
|  | 131 | 34 | DTC | USO | N | Y | 2y | Peritoneum, vaginal residue | TAH + RSO + omentectomy + appendectomy | chemotherapy | AWD at 7y |
| Li (2021) | 132 | 78 | FVPTC;  5cm | Partial pancreatectomy (metastasis of clear cell carcinoma of kidney), splenectomy; USO(LSO) | N | N | 40m | N | N | N | NED at 40m  (Normal thyroid gland on US/WBS) |
|  | 133 | 42 | PTC;  4cm | USO(LSO) | N | N | 5y | N | N | N | NED at 5y  (Normal thyroid gland on US) |
|  | 134 | 42 | PTC;  10cm | USO(LSO) | Chemotherapy | N | 3y | N | N | N | NED at 3y  (Normal thyroid gland on PET) |
|  | 135 | 56 | PTC;  5cm | USO, Debulking  (TAH + RSO + omentectomy + LN) | Chemotherapy | N | 70m | N | N | N | NED at 70m  (Normal thyroid gland on US) |
|  | 136 | 44 | PTC;  5.7cm;  primary PTC in neck | USO(LSO) | TT | N | 6m | N | N | N | NED at 6m  (TG undetectable) |
| Ayhan (2021) | 137 | 46 | FVPTC;  10cm | TAH + BSO + omentectomy + lymphadenectomy | TT, RAI | N | 0.83y | N | N | N | NED at 10m |
| Seban (2021) | 138 | 71 | PTC;  Primary PTC in neck | TAH + BSO | TT, RAI | Y | 0.5y | Liver, peritoneum | N | Targeted therapy and TACE | AWD at 22m |
| Yu (2021) | 139 | 42 | FTC;  7.8*7.7cm | USO (LSO) | Chemotherapy | Y | 2y | Uterus, peritoneum, mesentery and omentum | TAH + RSO + omentectomy + appendectomy + mesentery + peritoneal nodules resection | TT, RAI; chemotherapy; TACE | AWD at 18y |
| Cheng (2021) | 140 | 48 | PTC;  8.6*7.2cm | Right oophorectomy (USO) | N | N | 2y | N | N | N | NED at 2y |
| Donato (2021) | 141 | 35 | PTC; primary FVPTC in neck | Oophorectomy (USO) | TT, RAI | - | - | - | - | - | AWD at 2.4y |
| Our case | 142 | 46 | FTC; 5cm | TAH + BSO + omentectomy + pelvic lymphadectomy | chemotherapy | N | 12y | N | N | N | NED at 12y |

Abbreviations: MSO, malignant struma ovarii; PTC, papillary thyroid carcinoma; FTC, follicular thyroid carcinoma; FVPTC, follicular variant of papillary thyroid carcinoma; (D)TC, (differentiated) thyroid carcinoma; PDC, poorly differentiated thyroid carcinoma; USO, unilateral salpingo-oophorectomy; BSO, bilateral salpingo-oophorectomy; TAH/sTAH, total/subtotal abdominal hysterectomy; TT, total thyroidectomy; RAI, radioiodine therapy; EBRT, external beam radiotherapy; rh-TSH, recombinant human thyroid stimulating hormone; TACE, transcatheter arterial chemo-embolization; NED, no evidence of disease; AWD, alive with disease; DOD, die of the disease; NA, not applicable; R, recurrence; RFS, recurrent free survival.
