# Supplementary material for: Survival Outcomes and Prognostic Predictors in Patients With Malignant Struma Ovarii
Source: Front Med (Lausanne). 2021 Dec 23;8:774691. doi: 10.3389/fmed.2021.774691 (PMC8733601; doi:10.3389/fmed.2021.774691)
Supplement: Supplementary Table 1 — Database of our study (A) patients with malignant struma ovarii (MSO) confined to the ovary; (B) patients with metastatic MSO. [file Table_2.DOCX]

Table S1. Database of our study (patients with metastatic MSO).

| Reference | No. | Age(y) | Tumor size | pathology | Metastasis site | Surgery at metastasis | Adjuvant therapy | Results of follow-up |
| --- | --- | --- | --- | --- | --- | --- | --- | --- |
| Woodruff  (1966) | 1 | 52 | NA | FTC | liver | Oophorectomy  (USO) | radiotherapy | AWD at 7y |
| Kempers  (1970) | 2 | 59 | NA | FTC | Pelvic, lung | TAH + BSO | RAI | NED at 8y |
| Hasleton  (1978) | 3 | 73 | NA | FTC | Ileum, liver, omentum | NA | N | DOD at 2w later |
| Pardo-Mindan  Vasquez  (1983) | 4 | 60 | 17cm | Mxed PTC + FTC | Peritoneum, omentum;  Lungs, skin; lymph nodes | TAH + BSO | Chemotherapy, TT, EBRT | DOD at 2.5y |
| Willemse  (1987) | 5 | 36 | 10*10*6cm | FTC | Contralateral ovary;  Omentum;  peritoneum | BSO | TT, RAI | NED at 0.6y |
| Rosenblum  (1989) | 6 | 37 | NA | PTC | Contralateral ovary;  peritoneum | NA | chemotherapy | AWD at 4.5y |
| Mcdougall  (1989) | 7 | 42 |  | FTC | Bone (Throcic spine) | Metastatic lesion resection | TT, RAI | NED at 2y |
| Vadmal  (1997) | 8 | 48 | 5*4cm | FVPTC | Peritoneum,  Spleen, liver,  Diaphragmatic;  mesentery | Metastases resection | TT, RAI | NED at 1y |
| Tennvall  (1997) | 9 | 50 | 15cm | FTC | Omentum;  Bladder;  Peritoneum  (pouch of  Douglas) | sTAH + RSO + Omentectomy,  metastases resection;  (Debulking surgery) | TT, RAI | NED at 6y |
| Rotman-  Pikielny  (2000) | 10 | 46 | 20*18*17cm | FTC | Extensive liver metastasis | Ovarian cystectomy | TT, RAI, rhTSH | AWD at 0.5y |
| Chan  (2001) | 11 | 27 | NA | FTC | Bone (Thoracic spine) | N | TT, RAI | AWD at 15m |
| Kouraklis  (2001) | 12 | 52 | 13*11*8cm | TC | Ileum;  Retroperitoneum;  pelvic lymph nodes | TAH + BSO,  Metastatic lesion resection  (debulking) | TT, RAI | NED at 2y |
| Checrallah  (2001) | 13 | 42 | 12cm | TC | Pelvic bones and lung | N | TT, RAI, rhTSH | AWD at 2y |
| Mcdougall  (2006) | 14 | 58 | NA | TC | Liver, Bone  (lumbar spine) | N | TT, RAI | AWD at 6m |
| Roth &  Karseladze  (2008) | 15 | 58 | NA | FTC | Peritoneum; omentum; para-aortic lymph node | Metastases  resection | TT, RAI | AWD at 8y |
|  | 16 | 49 | NA | FTC | omentum | TAH+BSO | Chemotherapy | NED at 16y+4m |
|  | 17 | 50 | 15cm | FTC | omentum | TAH+RSO+ omentectomy (debulking) | TT, RAI | NED at 6y |
| Roth  (2008) | 18 | 26 | NA | PTC | paraaortic lymph nodes | Ovarian cystectomy +  metastases resection | radiotherapy | NED at 6y |
|  | 19 | 70 | 11*11*6cm | FTC  (poor differentiated) | Uterus;  Peritoneum | TAH + BSO | TT, RAI  chemotherapy | DOD at 3y |
| Salvatori  (2008)  Fabbri  (2018) | 20 | 22 | 6cm | FVPTC | peritoneum, diaphragm, vesical plica, and liver | (Fertility-preserving)  Ovarian cystectomy +  metastases resection | TT, RAI, rh-TSH | NED at 10y,  Transplantation of  cryopreserved ovarian failed |
| Yamashita  (2010) | 21 | 32 | NA | TC | Bone  (Lumbar spine) | Metastatic lesion resection | N | NED at 3y |
| Wolff  (2010) | 22 | 33 | 4cm | FVPTC | Peritoneum, ometnum, diaphragm, cecal and jejunal serosal nodules, spleen | TAH + BSO + Omentectomy  (debulking) | TT, RAI | AWD at 0.5y |
| Sibio  (2010) | 23 | 74 | 10cm | Brenner tumor  With PTC | peritoneum | TAH + BSO+  lymphadenectomy;  Metastatic lesion resection  (debulking) | N | NED at 7y |
| Minambres  (2011) | 24 | 38 | 8cm | FTC | Pelvic bone  (Right iliac bone), lung, lymph nodes | BSO;  (LSO + right oophorectomy) | TT, RAI | DOD at 14y |
| Steinman  (2013) | 25 | 22 | 9.5cm | PTC | Left anterior pelvis bone | USO | TT, RAI | NED at 5m |
| Ukita M  (2014) | 26 | 45 | 12cm | FTC | Lung, bone (rib, right scapula, left  Acetabulum) | USO;  (Left SO, wedge resection of right ovary) | chemotherapy | AWD at 24y |
| Carey  (2014) | 27 | 70 | NA | FTC;  PTC in thyroid | Peritoneum,  mediastinum | Debulking surgery | TT, RAI | AWD at 4m |
| Luo  (2014) | 28 | 46 | NA | PTC | Contralateral ovary, uterus, omentum, peritoneum | LSO, sequent debulking surgery  (TAH + RSO + omentectoy +  Appendectomy +  Pelvic and paraaortic  Lymph node resection) | TT, RAI | NED at 1y |
| Kobayashi  (2015) | 29 | 49 | NA | TC  (MSO) | Bone (Thoracic spine) | N | EBRT | NED at 9m |
| Seo  (2015) | 30 | 36 | NA | FTC  (Poor differentiated) | Liver;  Peritoneum | Peritoneal and hepatic metastases resection | TT, RAI;  chemotherapy | NED at 4y |
| Wei  (2015) | 31 | 39 | 10cm | FVPTC | Round ligamenm;  Liver, peritoneum (cul-de-sac) | NA | TT, RAI | AWD at 10y |
| Riaz  (2015) | 32 | 21 | 15cm | FVPTC | Omentum,  diaphragm, peritoneum, bone (skull, bilateral forearms), Lung | USO, omental  Biopsy | TT, RAI | AWD at 3m |
| Russo (2016) | 33 | 34 | 1.9cm | FVPTC | Contralateral Ovary;  Appendix;  Peritoneum; omentum | TAH + BSO + lymphadenectomy  (pelvic LN)  (debulking) | TT, RAI | NED at 8.6y |
| Zhu (2016) | 34 | 40 | 3cm | PTC | omentum | TAH + BSO + omentectomy + appendectomy + LN  (debulking) | Chemotherapy (TC*6) | NED at 1y |
| Anagnostou  (2016) | 35 | 64 | 13cm | FTC | Peritoneum  (the pouch of Douglas) | TAH + BSO + omentectomy  and appendectomy  (debulking) | N | NED at 4y |
| Williams  (2016) | 36 | 61 | 25cm | PTC mixed  With  Insular  carcinoma | uterine serosa | TAH + BSO + metastases resection + omentectomy +  rectosigmoid resection + anastomosis (debulking) | TT | NED at 3m |
| Oudoux  (2016) | 37 | 67 | 5cm | PTC | retrocaval lymph node, omentum | TAH + BSO +  Omentectomy + LN  (debulking) | TT, RAI | NED at 5y |
| Comunello  (2017) | 38 | 38 | 5.2cm | FTC | Bone  (Left scapula) | USO, partial scapulectomy | TT, RAI | NED at 1y  (2y?) |
| Gobitti  (2017) | 39 | 36 | 10cm | FVPTC | Peritoneum;  Liver; uterus | TAH + BSO + Omentectomy + appendectomy + hepatic nodule resection;  (debulking) | TT, RAI, rh-TSH | AWD at 3m |
| Ernaga  (2018) | 40 | 57 | 10cm | FVPTC; TC  (Poor differentiated) | Contralateral ovary; Liver;  Peritoneum | BSO | TT, RAI  Chemotherapy | DOD at 18m |
|  | 41 | 48 | 10cm | FVPTC | Peritoneum | TAH + BSO + metastases resection  (debulking) | TT, RAI | NED at 3y |
| Lager (2018) | 42 | 30 | 8cm | PTC | Bone (Rib;  proximal femurs;  sacrum); lung | USO | TT, RAI | AWD at 9m |
| Seifert (2019) | 43 | 67 | 4.4cm | FVPTC | Bone (Femoral shaft) | BSO | RAI | NED at 30m |
| Szczepanek  (2019) | 44 | 19 | 6cm | FVPTC | Pelvic bone  (Left ilium) | USO | TT, RAI | NED at 9y |
| Tsukada (2019) | 45 | 39 | 7cm | FTC | omenteum | TAH + BSO + omentectomy (debulking) | TT, RAI*4 | AWD at 2y |
| Gild  (2020) | 46 | 33 | NA | FTC | Bone  (Scapula, Rib) | Scapular metastasis resection | TT, RAI | NED at 2y |
| Li （2020） | 47 | 38 | 15cm | FTC | Uterus, omentum, liver, lung | debulking surgery (TAH + BSO + omentectomy + pelvic lymph nodes dissection) | TT, RAI | AWD at 5y |
|  | 48 | 33 | 4cm | DTC | Pelvic bone  (Left acetabulum), peritoneum | Left ovarian cystectomy, metastases resection, bone biopsy | N | AWD at 15m |
| Tang (2020) | 49 | 27 | 20cm | FTC | Contralateral ovary and peritoneum | TAH + BSO + appendectomy (debulking); laparoscopic biopsy at recurrence | Chemotherapy; TT, RAI | 1^st^ recurrence at 21y and NED at 33y |
| Chiang (2021) | 50 | 43 | 12cm | FTC | Right fallopian tube, infundibulopelvic fold, bladder, rectal wall, and peritoneum | TAH + BSO + omentectomy (debulking) | TT, RAI | NED at 1y |
| Chung (2021) | 51 | 62 | 2cm | FTC | Liver, lung | Plural biopsy and wedge resection of the right lower lobe | TT, RAI; TKI targeted therapy | AWD at 5y |
| Taelman (2021) | 52 | 82 | 22*14*10cm | PTC | Liver | USO, biopsy of liver and lymph nodes | TT, RAI | AWD at 14y |

Abbreviations: MSO, malignant struma ovarii; PTC, papillary thyroid carcinoma; FTC, follicular thyroid carcinoma; FVPTC, follicular variant of papillary thyroid carcinoma; (D)TC, (differentiated) thyroid carcinoma; USO, unilateral salpingo-oophorectomy; BSO, bilateral salpingo-oophorectomy; TAH/sTAH, total/subtotal abdominal hysterectomy; TT, total thyroidectomy; RAI, radioiodine therapy; EBRT, external beam radiotherapy; rh-TSH, recombinant human thyroid stimulating hormone; NED, no evidence of disease; AWD, alive with disease; DOD, die of the disease; NA, not applicable; R, recurrence; RFS, recurrent free survival.
