# Supplementary material for: Survival Outcomes and Prognostic Predictors in Patients With Malignant Struma Ovarii
Source: Front Med (Lausanne). 2021 Dec 23;8:774691. doi: 10.3389/fmed.2021.774691 (PMC8733601; doi:10.3389/fmed.2021.774691)
Supplement: Supplementary Table 2 — The univariate and multivariate analysis of clinical outcomes. [file Table_3.DOCX]

**Table S2** Univariate and multivariate analysis of clinical outcomes

| Factors | NED | AWD/DOD | X^2^ | P value | Logistic regression analysis |
| --- | --- | --- | --- | --- | --- |
|  |  |  |  |  | OR (95%CI) P |
| Age (<45/>=45, years) | 66/80 | 26/22 | 1.163 | 0.281 |  |
| FIGO stage ^a,^ *  Stage I/ Stage II-III  Stage I/ Stage IV  Stage II-III/ Stage IV | 120/13  120/13  13/13 | 22/5  22/5  5/21 | 31.641 | <0.001* | <0.001  1.524 (0.452, 5.135) 0.497  7.328 (3.103, 16.885) <0.001*  4.750 (1.264, 17.856) 0.021* |
| Follicular carcinoma subtype (Yes/No) ^a^  poorly differentiated (Yes/No) | 35/111  5/141 | 19/29  4/44 | 4.382  1.967 | 0.036*  0.229 |  |
| Tumor size (<8/>=8, cm) | 56/39 | 7/19 | 8.388 | 0.004* |  |
| Surgical options ^a^ |  |  | 5.980 | 0.036* |  |
| No surgery/conservative surgery | 1/68 | 3/26 |  |  |  |
| No surgery/aggressive surgery | 1/61 | 3/15 |  |  |  |
| Conservative surgery/aggressive surgery | 68/61 | 26/15 |  |  |  |
| RAI therapy (Yes/No) ^a^ | 27/95 | 22/25 | 2.863 | 0.091 |  |

Abbreviations: RAI, radioiodine therapy; NED, no evidence of disease; AWD, alive with disease; DOD, die of the disease.

*a* Factors applied to multivariate analysis; * *p* < 0.05*
