# Supplementary material for: Survival Outcomes and Prognostic Predictors in Patients With Malignant Struma Ovarii
Source: Front Med (Lausanne). 2021 Dec 23;8:774691. doi: 10.3389/fmed.2021.774691 (PMC8733601; doi:10.3389/fmed.2021.774691)
Supplement: Supplementary Table 3 — The univariate and multivariate analysis of overall survival (OS). [file Table_4.DOCX]

**Table S3** Univariate and multivariate analysis of OS

| Factors | N |  | Univariate analysis | | |  | Multivariate cox regression analysis | | | |
| --- | --- | --- | --- | --- | --- | --- | --- | --- | --- | --- |
|  |  | Mean survival(y) | | 5-year survival rate | p |  | OR | (95% CI) | | p |
| Age (<45/>=45, years) ^a^ | 92/102 | 37.7/21.8 | | 100%/85.1% | 0.022 |  | 4.959 | | 1.093-22.508 | 0.038 |
| FIGO stage  Stage I/ Stage II-III  Stage I/ Stage IV  Stage II-III/ Stage IV | 142/18  142/34  18/34 | 36.4/30.5  36.4/18.0  30.5/18.0 | | 92.6%/91.7%  92.6%/86.1%  91.7%/86.1% | 0.876  0.131  0.331 |  |  | | | |
| Follicular carcinoma subtype (Yes/No)  poorly differentiated (Yes/No) ^a, *^ | 54/140  9/185 | 27.4/36.4  6.0/36.4 | | 92.7%/90.5%  70.0%/92.7% | 0.679  0.001 |  | 6.406 1.730-23.717 0.005 | | | |
| Tumor size (<8/>=8, cm) | 63/58 | 24.2/26.4 | | 98.4%/84.7% | 0.197 |  |  | | | |
| Surgical options |  |  |  | | |  |  | | | |
| No surgery/conservative surgery | 4/94 | - | | -/92.7% | 0.862 |  |  | | | |
| No surgery/aggressive surgery | 4/76 | - | | -/88.9% | 0.787 |  |  | | | |
| Conservative surgery/aggressive surgery | 94/76 | - | | 92.7%/88.9% | 0.446 |  |  | | | |
| RAI therapy (Yes/No) | 69/120 | 24.4/33.4 | | 90.8%/91.3% | 0.832 |  |  | | | |

a, Factors applied to multivariate analysis; -, Not available; *, p < 0.05

Abbreviations: RAI, radioiodine therapy; OS, overall survival.
