# Supplementary material for: Survival Outcomes and Prognostic Predictors in Patients With Malignant Struma Ovarii
Source: Front Med (Lausanne). 2021 Dec 23;8:774691. doi: 10.3389/fmed.2021.774691 (PMC8733601; doi:10.3389/fmed.2021.774691)
Supplement: Supplementary Table 4 — The univariate and multivariate analysis of disease-specific survival (DSS). [file Table_5.DOCX]

**Table S4** Univariate and multivariate analysis of DSS

| Factors | N |  | Univariate analysis | | |  | Multivariate cox regression analysis | | | |
| --- | --- | --- | --- | --- | --- | --- | --- | --- | --- | --- |
|  |  | Mean survival(y) | | 5-year survival rate | p |  | OR | (95% CI) | | p |
| Age (<45/>=45, years) ^a^ | 92/102 | 37.7/22.7 | | 100%/89.3% | 0.081 |  |  | |  |  |
| FIGO stage ^a^  Stage I/ Stage II-III  Stage I/ Stage IV  Stage II-III/ Stage IV | 142/18  142/34  18/34 | 37.6/30.5  37.6/18.0  30.5/18.0 | | 95.9%/91.7%  95.9%/86.1%  91.7%/86.1% | 0.082  0.783  0.027  0.331 |  |  | | | |
| Follicular carcinoma subtype (Yes/No)  poorly differentiated (Yes/No) ^a, *^ | 54/140  9/185 | 27.4/37.7  6.0/37.3 | | 92.7%/94.1%  70.0%/95.3% | 0.270  < 0.001 |  | 9.664 2.409-38.760 0.001 | | | |
| Tumor size (<8/>=8, cm) | 63/58 | 24.1/20.4 | | 100%/88.2% | 0.140 |  |  | | | |
| Surgical options |  |  |  | | |  |  | | | |
| No surgery/conservative surgery | 4/94 | - | | -/96.4% | 0.903 |  |  | | | |
| No surgery/aggressive surgery | 4/76 | - | | -/90.1% | 0.886 |  |  | | | |
| Conservative surgery/aggressive surgery | 94/76 | - | | 96.4%/90.1% | 0.287 |  |  | | | |
| RAI therapy (Yes/No) | 69/120 | 24.4/34.6 | | 94.8%/90.8% | 0.349 |  |  | | | |

a, Factors applied to multivariate analysis; -, Not available; *, p < 0.05

Abbreviations: RAI, radioiodine therapy; DSS, disease-specific survival.
